# Palm sEMG-based user identification during doorknob rotation using a convolutional neural network

**DOI:** 10.1038/s41598-026-46294-3

**Published:** 2026-05-16

**Authors:** Yeonjung Shin, Junghun Kim, Sang-Il Choi

**Affiliations:** 1 Department of Computer Software, Daegu Catholic University, Gyeongsan-si, Gyeongsangbuk-do 38430 Republic of Korea; 2 School of Computer Software, Daegu Catholic University, Gyeongsan-si, Gyeongsangbuk- do 38430 Republic of Korea

**Keywords:** Deep learning, sEMG, Identification, Engineering, Mathematics and computing, Neuroscience

## Abstract

Convenient and secure user identification is increasingly important in everyday environments, particularly with the proliferation of contactless interactions and Internet-of-Things (IoT) devices. However, conventional authentication methods often require explicit user input or additional hardware, limiting their usability in natural daily scenarios. To address this issue, we propose a doorknob-rotation-based user identification method using palm surface electromyography (sEMG). sEMG signals were acquired from the abductor pollicis brevis and abductor digiti minimi at 1,000 Hz, denoised using a 60 Hz notch and 20–500 Hz band-pass filters, and transformed into time–frequency spectrograms via continuous wavelet transform. A DenseNet161 model was employed for classification. Using data from five participants, the proposed method achieved 94.00% test accuracy and 93.99% F1-score, with five-fold cross-validation accuracy of 91.66$\:\pm\:$2.78%. The approach enables on-device, contact-based identification without wireless pairing, transforming everyday actions into seamless authentication. These results demonstrate the feasibility and practical potential of sEMG-based everyday-action user identification.

## Introduction

The rise of the information society, the recent proliferation of Internet-of-Things(IoT) devices, the normalization of remote work environments, and advances in smart healthcare systems highlight the importance of technologies that can provide secure and rapid user identification anytime and anywhere^1,2^. Commercially deployed user identification methods are commonly categorized as knowledge-based, possession-based, and biometric: these methods rely on information remembered by the user (e.g., passwords and personal identification numbers(PINs)), possession-based identification relies on physical tokens carried by the user (e.g., smart cards or security tokens), and a user’s unique physiological traits (e.g., fingerprints, the iris, or the face), respectively^3–5^. However, knowledge-based methods are vulnerable to theft or leakage, possession-based items can be lost or forged, and although biometric identification offers high convenience and security, externally exposed biometric traits such as fingerprints and faces can be spoofed. Moreover, once fixed biometric identifiers are compromised, they cannot be changed, which is a critical limitation^6–8^. Consequently, new user identification approaches based on dynamic biometric signals have attracted increasing attention.

Dynamic biometric signals reflect a user’s physiological and behavioral characteristics that can exhibit subtle variations at each measurement. Representative examples include electrocardiography (ECG), electroencephalography (EEG), and electromyography (EMG). Such signals are difficult to counterfeit or imitate from outside the body and vary with context and behavior, thereby addressing the vulnerabilities inherent to fixed biometrics. Among these, surface (s)EMG measures the electrical activity generated during muscle contraction and relaxation using electrodes attached to the skin surface. Because sEMG enables non-invasive measurement of muscular activity, it can be integrated with wearable devices to implement real-time identification systems^7,9^. In particular, the amplitude and spectral content of sEMG are closely related to the degree of activation, fatigue, and contraction speed of muscles, and they also embed individual-specific properties arising from muscle morphology and neural innervation patterns. As a result, sEMG patterns are inherently user-specific and difficult to reproduce externally, which mitigates spoofing. Moreover, when a user performs a specific action, the resulting sEMG pattern exhibits high reproducibility and clear separability from other actions, and can be realized in the form of arm or wrist bands. These characteristics enable seamless integration of identification into daily routines, motivating active research in this area^10,11^.

Recent sEMG-based user identification studies can be broadly divided into gesture-based and rest (no-motion)–based paradigms. Gesture-based user identification extracts sEMG patterns while the user performs pre-registered actions and uses them for identification. Rest-based user identification analyzes subtle muscular activity during quiescence or th maintenance of a specific posture. Researchers have applied diverse feature-extraction methods and a range of machine-learning and deep-learning algorithms, including support vector machine (SVM), random forest, k-nearest neighbor (k-NN), and convolutional neural network (CNN), with reportedly high classification accuracy^12,13^.

Most existing sEMG-based user identification studies target relatively simple actions such as finger flexion, wrist rotation, and fist clenching, and these actions share the common premise that they are gestures intentionally performed for the purpose of authentication or identification. Although such approaches are advantageous for validating algorithmic performance, they may hinder a natural user experience in real-world environments, as users must perform a separate action specifically for identification. In addition, pipelines based on fine hand movements that are validated in experimental settings have limitations when directly applied to complex movements that repeatedly occur in everyday environments.

To address this limitation, the present study reinterprets doorknob rotation, an everyday and repetitive natural behavior, as a user identification trigger. The act of opening a door is performed without conscious awareness of a specific system, and the palm muscle activation patterns generated during this process reflect an individual user’s unique muscle usage habits and force distribution. Therefore, this study differs from prior work in that it goes beyond gestures-based user identification and proposes a new problem definition that utilizes everyday behavior itself as a cue for user identification.

Furthermore, many existing studies are based on wearable devices with sensors attached to the forearm or wrist, assuming external devices such as smartwatches or armbands and wireless connections. In contrast, this study experimentally verified that user identification is possible using only palm sEMG without using the wrist or forearm at all. By assuming a scenario in which the sensor directly contacts the target device, user identification can be achieved without separate wearable devices or Bluetooth/Wi-Fi pairing. This approach reduces system complexity while suggesting the potential for natural integration into real physical interfaces.

The problem setting addressed in this study is closed-set user identification, in which the system aims to determine which registered user corresponds to the input sEMG signal within a predefined user set. The data collected in this study are structured as a multi-class configuration with explicit user labels for each participant, and all evaluations were conducted under the assumption of this enrolled user set. This setting aligns not only with experimental conditions but also with practical application scenarios in which a limited group of users repeatedly interacts with the same physical interface.

In summary, the novelty and main contributions of this study are as follows. First, this study introduces doorknob rotation, an everyday and naturally repeated action, as a user identification cue, extending sEMG-based identification beyond intentionally performed gestures. Second, it demonstrates that user identification is feasible using only two-channel palm sEMG, without relying on wrist/forearm wearables or wireless pairing. Third, it validates a practical CWT–DenseNet161 pipeline and shows superior performance over multiple baseline models under the same experimental setting.

## Related work

Recent studies have further advanced sEMG gesture recognition through multi-stream architectures, time-varying feature enhancement, and CNN optimization, reporting meaningful performance improvements on multi-channel sEMG datasets^14,15^. In addition, non-CNN time-series methods such as Random Convolutional Kernel Transform (ROCKET) have also demonstrated competitive performance in hand/wrist sEMG classification, indicating that they can serve as complementary baselines to deep CNN approaches^16^(Table 1).

Buriro et al.^17^ proposed a dual-modal behavior-based user identification system that combines forearm motion signals and wrist sEMG in a smartwatch environment. Data were collected from 50 participants during hand clapping tasks, and by simultaneously utilizing both modalities, it achieved higher discriminative power compared to single-biometric approaches, and reported superior performance in both true acceptance rate (TAR) and false acceptance rate (FAR) through GAN-based data augmentation. These results demonstrate that the integration of multimodal information can effectively enhance the robustness of identification systems. However, the system configuration is complex and depends on a specific wearable environment, which requires additional consideration for extension to various devices and environments.

Fan et al.^18^ proposed a user authentication method for smartphone unlocking using forearm sEMG signals and directly learned inter-user similarity through a CNN-based Siamese network architecture. The Siamese structure is suitable for authentication problems because it can effectively model signal variability across users, and its applicability in mobile environments was experimentally validated using data collected from 80 participants during smartphone unlocking tasks. However, it assumes the use of a forearm wearable device, and the usage environment may be restricted depending on the integration structure with the target device.

Gursoy et al.^19^ proposed a hand gesture-based sEMG user identification system that combines various wavelet-based feature extraction techniques (discrete wavelet transform(DWT), empirical wavelet transform(EWT), empirical mode decomposition(EMD)) with CNN. Based on data collected from 5 participants performing six hand gestures, EMD-based features effectively reflected the non-stationary characteristics of sEMG signals and achieved high classification performance. This study demonstrated that frequency decomposition-based approaches are meaningful for capturing user-specific muscle activation patterns; however, the multi-stage signal processing pipeline increases the complexity of system implementation and parameter configuration, which requires consideration from a practical perspective.

Kim et al.^20^ combined constant-Q transform(CQT)-based two-dimensional spectrogram representation with a CNN classifier and reported improved user identification performance compared to conventional one-dimensional time-domain features and short-time Fourier transform(STFT)-based baselines. Based on data obtained from the NinaPro DB2 dataset consisting of 40 participants performing hand gesture tasks, CQT has the advantage of adaptively adjusting resolution according to frequency bands, thereby effectively reflecting the nonlinear characteristics of sEMG signals and inter-individual differences in frequency response. However, since it requires multi-channel upper-arm muscle signals, there remains room for simplification in terms of sensor placement and system configuration.

Lu et al.^21^ converted forearm sEMG signals into continuous wavelet transform(CWT)-based time–frequency spectrograms and combined CNN with a Siamese network to maximize user recognition performance. Based on data collected from 21 participants performing hand opening tasks, this study demonstrated that combining time–frequency representation with deep learning-based similarity learning can effectively model the individual specificity of sEMG signals. However, due to the forearm-centered sensor placement and task-specific experimental settings, performance variability may occur depending on the application environment.

Most existing studies acquire sEMG signals from the forearm or wrist and combine various time–frequency representations such as CQT, DWT, and CWT with CNN-based models, achieving accuracies of 95–99%. Time–frequency transformations such as CWT and CQT have been proven effective for the non-stationary characteristics of sEMG. However, existing studies have primarily used limited hand gestures suitable for laboratory environments and wrist/forearm-centered sensor placement, which constrains the direct application of the technology to everyday behavior-based user identification in real-world environments.

**Table 1:** Comparison of prior sEMG-based user identification studies.

Muscle site	Participants	Ch.*	Action	Feature extraction	Model	Accuracy (%)	References
Wrist	50	8	Hand clapping	GAN	DNN	97.94	^17^
Forearm	80	8	Smartphone unlocking	–	Siamese CNN	92.06	^18^
Forearm	5	4	Six hand gestures	DWT, EWT, EMD	CNN	up to 95.62	^19^
Biceps, triceps	40	12	Three hand gestures	CQT	CNN	97.5	^20^
Forearm	21	4	Hand opening	DWT, CWT	CNN	up to 99.206	^21^

*Number of channels.

Prior studies that evaluated the representational capacity and generalization performance of various deep learning architectures on sEMG signals, not limited to the purpose of user identification, are summarized (Table 2). Although these studies primarily aimed at hand gesture recognition or classification of specific muscle activation patterns, they provide important implications for the present study from the perspective of network design for effectively modeling the nonlinearity and temporal variability of sEMG signals.

The CNN–BiLSTM-based approach proposed by Kishore et al.^22^ effectively extracts local spatial features of sEMG signals through CNN and then models temporal dependencies bidirectionally using BiLSTM, thereby enabling precise learning of differences in muscle activation patterns among users. In particular, by simultaneously utilizing contextual information from past and future time steps, it can stably capture user-specific time-series characteristics even within repeatedly performed actions. Due to these characteristics, the CNN–BiLSTM architecture is suitable as a baseline model for handling signals with large temporal variability in sEMG-based user identification problems.

The CNN–LSTM–Attention architecture of Hwang et al.^23^ combines an attention mechanism with LSTM-based temporal modeling, enabling selective emphasis on muscle activation features in time segments that are important for identification for each user. This structure reflects the fact that not all time segments within sEMG signals contain the same identification information, and is effective in highlighting subtle differences in activation patterns among users. For this reason, the model is meaningful as a comparison baseline for analyzing the impact of temporal importance learning on user identification performance.

The E2CNN proposed by Qureshi et al.^24^ is a CNN architecture that utilizes Log-Mel-based spectrogram input and a concatenation structure to simultaneously achieve high classification performance and efficient feature learning with a relatively small number of parameters. The approach of combining multi-level features from the input stage is advantageous for reflecting various representations of sEMG signals without loss and has structural advantages in stably learning differences in muscle activation patterns among users. Accordingly, E2CNN was selected as a representative CNN-based baseline model for simultaneously evaluating performance and efficiency in the user identification problem.

The Tri-CCNN proposed by Katherine et al.^25^ provides richer feature representations compared to single-input models by learning sEMG representations extracted in the time–frequency domain in parallel using a multi-stream CNN structure. The multi-stream architecture can simultaneously reflect different time–frequency characteristics for each user, thereby contributing to the formation of clearer identification boundaries among users. Due to these characteristics, Tri-CCNN is suitable as a model for comparing and analyzing the impact of time–frequency-based representation learning on user identification performance.

In this way, studies that evaluated the feature learning capability and generalization performance of various deep learning architectures on sEMG signals, not limited to user identification, mainly utilized CNN, LSTM, attention mechanisms, and multi-stream structures to effectively model the spatial, temporal, and time–frequency characteristics of sEMG signals, and reported high representational capacity capable of distinguishing differences in muscle activation patterns among users. In particular, time-series enhanced architectures such as CNN–BiLSTM and CNN–LSTM–Attention, as well as structural feature combination models such as E2CNN and Tri-CCNN, demonstrated that they can stably reflect the non-stationarity and temporal variability of sEMG signals. Although the primary objective of these studies was action or gesture classification, they share the common characteristic of learning differences in muscle activation patterns among users, thereby providing important implications as baseline models applicable to sEMG-based user identification problems.

**Table 2:** Comparison of deep learning models for sEMG-based studies.

Muscle site	Participants	Ch.*	Action	Feature extraction	Model	Accuracy (%)	References
Forearm	40	12	50 hand/finger movements	–	CNN-BiLSTM	96.45	^22^
lower limb	35	6	walking/ Climbing stairs	–	CNN-LSTM-Attention	Up to 98.41	^23^
Forearm	27	6	eleven hand gestures	LMS	E2CNN	up to 98.31	^24^
Flexor digitorum profundus	20	1	fist grasp	TFR	Tri-CCNN	93.33	^25^

*Number of channels.

### Methods

**Fig. 1:**
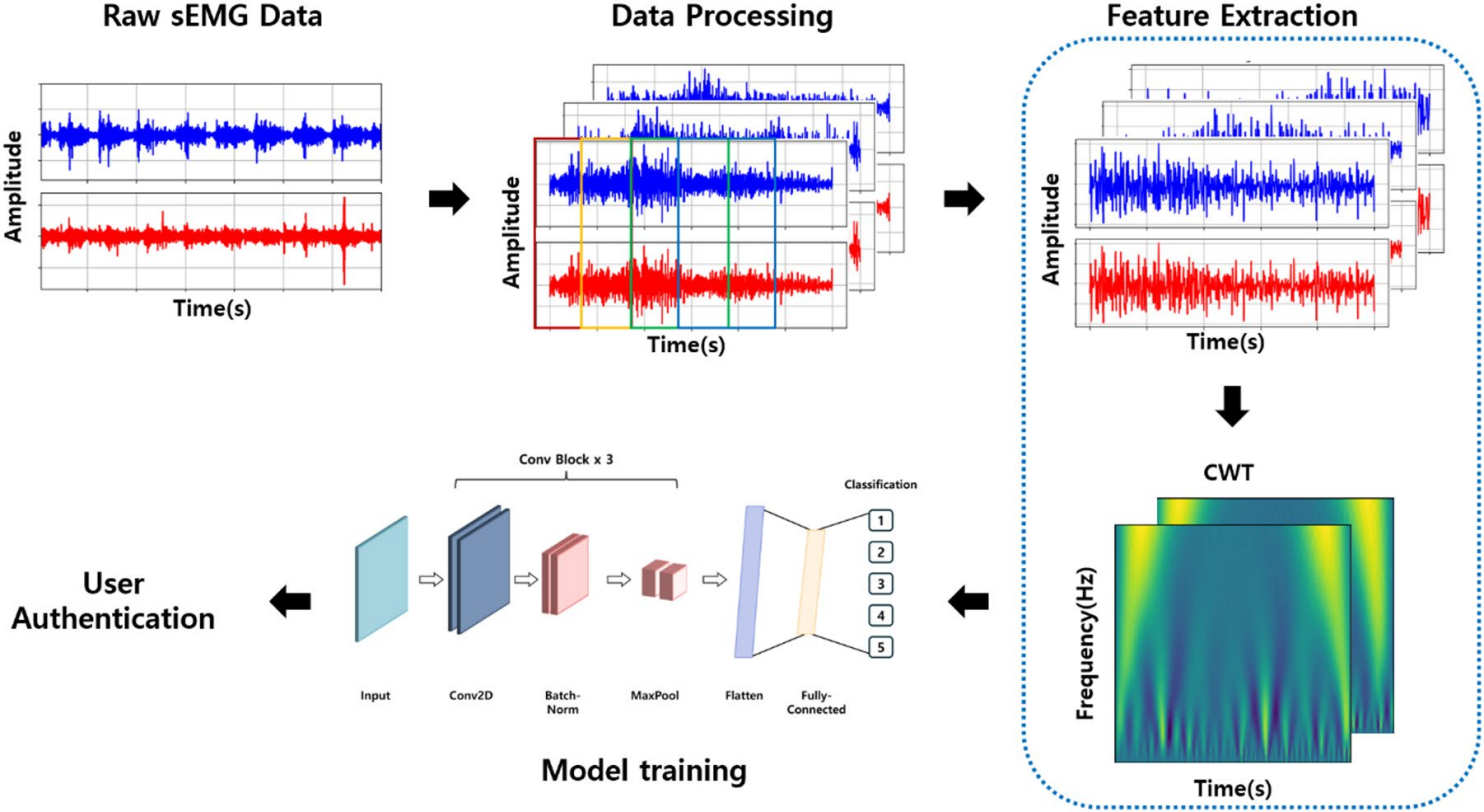
Overall architecture of the proposed sEMG-based user identification system.

The overall architecture of the system proposed in this study consists of sEMG data acquisition, a data preprocessing process, a 2D spectrogram conversion process, and a final identification process via a CNN model (Fig. 1). Raw sEMG signals are acquired through electrodes attached to the palm, after which filters are applied for noise removal. The preprocessed signals are segmented using a sliding window, and CWT is performed for each segment to convert them into two-dimensional time–frequency spectrograms. The generated spectrograms are used as input to the CNN model, and ultimately, the user corresponding to the input signal is identified within the registered user set.

To remove power-line interference from the raw sEMG signals, a 60 Hz notch filter was applied, followed by a band-pass filter that passes the 20–500 Hz frequency band to preserve components related to muscle activity. The denoised sEMG signals were labeled as a single action and then segmented by applying a sliding window to the signals. A 300 ms window length was set to sufficiently capture the temporal patterns of the electromyographic signals while preventing excessive data redundancy, and a 50% overlap was used to secure temporal continuity between adjacent sections, thereby minimizing specific losses while increasing the number of data samples to improve training stability. Each segment was scaled to between 0 and 1 through min–max normalization to eliminate differences in signal magnitude between channels and to increase convergence speed and performance stability during model training.

### Time–frequency feature representation

In this study, CWT was used as the sEMG feature-extraction method. sEMG is a biosignal that reflects the electrical activity associated with muscle contraction and relaxation, and it generally exhibits non-stationary characteristics, with frequency components that change rapidly over time. These characteristics have an important impact on the choice of analysis method.

Traditional time-domain features such as mean absolute value, root mean square, and integrated EMG can intuitively represent the amplitude, energy, and variability of the signal and have the advantage of being suitable for real-time applications due to their low computational cost. However, time-domain features alone cannot directly analyze the frequency distribution or its changes in the signal, which leads to limitations in finely distinguishing dynamic movements or transient muscle activation patterns. In addition, frequency domain features are advantageous for analyzing the overall frequency components of the signal and the energy distribution of specific bands. However, because time information is not considered in frequency domain features, it is difficult to determine how frequency changes occur over time. These limitations can lead to degraded performance in sEMG analysis, where time-dependent characteristics such as transient muscle activation changes or action transitions are important. To overcome these limitations, we utilized CWT (a time–frequency analysis technique). By analyzing the signal at multiple scales, CWT provides higher resolution in the low-frequency region and higher resolution in the high-frequency region, thereby effectively reflecting the non-stationary characteristics of sEMG signals^26,27^.

The definition of CWT used in this study is given by Eq. (1).

1 $$\:{CWT}_{x}\left(a,b\right)=\frac{1}{\sqrt{\left|a\right|}}{\int\:}_{-\infty\:}^{\infty\:}x\left(t\right){\psi\:}^{*}\left(\frac{t-b}{a}\right)dt,$$

where $\:\mathrm{x(t)}$ is the preprocessed sEMG signal, $\:{\psi\:}^{*}$ is the mother wavelet, scale parameter $\:a$ corresponds to high-frequency analysis with a short time window when $\:a$ is small and to low-frequency analysis with a long time window when $\:a$ is large, and time-shift parameter $\:b$ determines the position of the analysis window over the entire signal. CWT enables the selection of various mother wavelets to perform scale-specific transforms tailored to the characteristics of the signal, thereby enabling simultaneous analysis of local patterns and transient events. In the case of sEMG signals, this multi-scale analysis can effectively distinguish between abrupt muscle contractions and relaxations, frequency shifts due to fatigue accumulation, and subtle differences in motion.

Accordingly, in this study, the sEMG signals transformed through CWT were configured as input tensors in the form of time–frequency spectrograms. The CWT results were converted into a two-dimensional representation of size 3 × 32 × 300 and used as input to the CNN. Here, 32 denotes the number of scales applied during the CWT transformation, which was set to sufficiently reflect the major frequency components of the sEMG signals while avoiding excessive computational complexity. In addition, 300 corresponds to the length of the time axis for a 300 ms sliding window and was determined to stably include the muscle activation change patterns that appear during a single action execution. The value 3 represents the input channel dimension, configured to match the CNN input format after converting the sEMG signals measured from the palm into time–frequency image form. This input configuration effectively represents the non-stationary characteristics of sEMG signals in the time–frequency domain while considering the balance between model training stability and computational efficiency.

### The proposed model

In this study, the time–frequency spectrum generated through CWT was converted into a 2D image subsequently used as the input to a CNN. This enabled the CNN to learn spatially adjacent time–frequency patterns and to distinguish even subtle differences between actions.

The proposed model in this study is based on the DenseNet architecture^28^. Unlike conventional CNN architectures, dense connections are adopted that directly connect the outputs of all layers to subsequent layers; i.e., each layer can use the feature maps of all preceding layers as input rather than simply receiving the output of the immediately previous layer, thereby maximizing information flow within the network. DenseNet consists of dense blocks and transition layers. A dense block is composed of multiple convolutional layers, with each layer using the outputs of the preceding layers concatenated along the channel dimension as its new input. In this way, the network integratively learns rich multi-level features as the depth increases. A transition layer consists of a 1 × 1 convolution and 2 × 2 average pooling, which reduces the number of channels and halves the spatial size to improve computational efficiency.

Among the DenseNet family, we adopted DenseNet161 configured with dense blocks of [6, 12, 36, 24] and a growth rate of 48, which is larger than in other versions. This means that the four Dense Blocks consist of 6, 12, 36, and 24 bottleneck layers, respectively, and 48 new feature maps are generated at each layer. The input data (3 × 32 × 300) pass through an initial 7 × 7 convolution (stride = 2) and a 3 × 3 max pooling layer, during which the spatial resolution is progressively reduced. Subsequently, as the data pass through each Dense Block and Transition Layer, the number of channels increases while the spatial dimensions decrease step by step. The output of the final Dense Block is converted into a single 2208-dimensional feature vector through Global Average Pooling, and is then mapped to an output corresponding to the final number of user classes through a fully connected layer.

This has the advantage that each block generates many feature maps, thereby enabling fine discrimination of subtle differences in signal patterns. In particular, for data such as palm sEMG signals (where the input size is small and frequency changes are subtle), DenseNet161 can stably learn complex time–frequency patterns through a balance of depth and width.

**Fig. 2:**
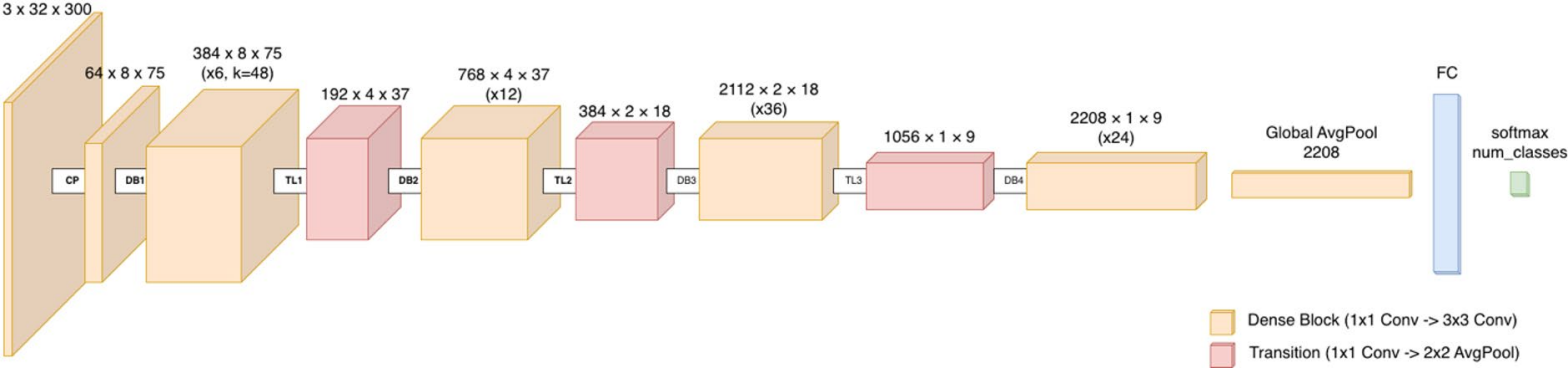
Diagram of the proposed DenseNet161 model.

In the present study, we based our implementation on DenseNet161 provided by PyTorch, and the input data were 2D time–frequency spectrograms generated by converting sEMG signals measured on the palm using CWT (Fig. 2). The first convolutional layer and the output layer were modified to match the input size, and dropout was applied to prevent overfitting. The Adam optimizer was used for model training, with a learning rate set to 0.001, a batch size of 16, and the number of training epochs set to 45. The cross-entropy loss function was used as the loss function. In addition, pre-trained weights were not used during model training, and all network parameters were learned from scratch through random initialization. This choice was made in consideration of the fundamentally different data distributions and statistical characteristics between natural images and sEMG-based CWT images. Furthermore, by excluding the influence of transfer learning, we aimed to more clearly and fairly analyze the impact of the proposed model architecture and the sEMG signals themselves on user identification performance.

### Data collection from participants

Our aims were to evaluate the feasibility of user identification using sEMG signals and to optimize the approach by comparing the performances of various model architectures, with the primary objective placed on validating the proposed pipeline (data preprocessing, feature extraction, and classification) rather than verifying its generalizability to a large population. Following prior studies, with priority placed on validating the feasibility of the proposed method, the initial experiments were conducted with a limited group of five participants^19,29,30^. 

The cohort was designed so that some physiological factors (age and body condition) would be similar and signals could overlap, thereby confirming that the proposed model can stably distinguish inter-individual signal differences even under similar conditions. The participants consisted of two females and three males, with a mean age of 25$\:\pm\:$2 years. The female participants were 169$\:\pm\:$1 cm tall and 59$\:\pm\:$6 kg in weight (i.e., within a relatively similar body type), whereas the male participants were 180$\:\pm\:$5 cm tall and 80$\:\pm\:$11 kg in weight. By composing the experimental group within a similar age range and body conditions, the study focused on verifying that clear user discrimination is possible even among similar signals. In general, differences in age, body type, and muscle mass may affect the amplitude and frequency characteristics of sEMG signals, and when such differences are large, the model may rely on relatively distinct physiological differences to distinguish users. Therefore, in this study, we aimed to minimize the influence of such external factors and to evaluate the possibility of identification based on more subtle differences in muscle activation patterns.

**Fig. 3:**
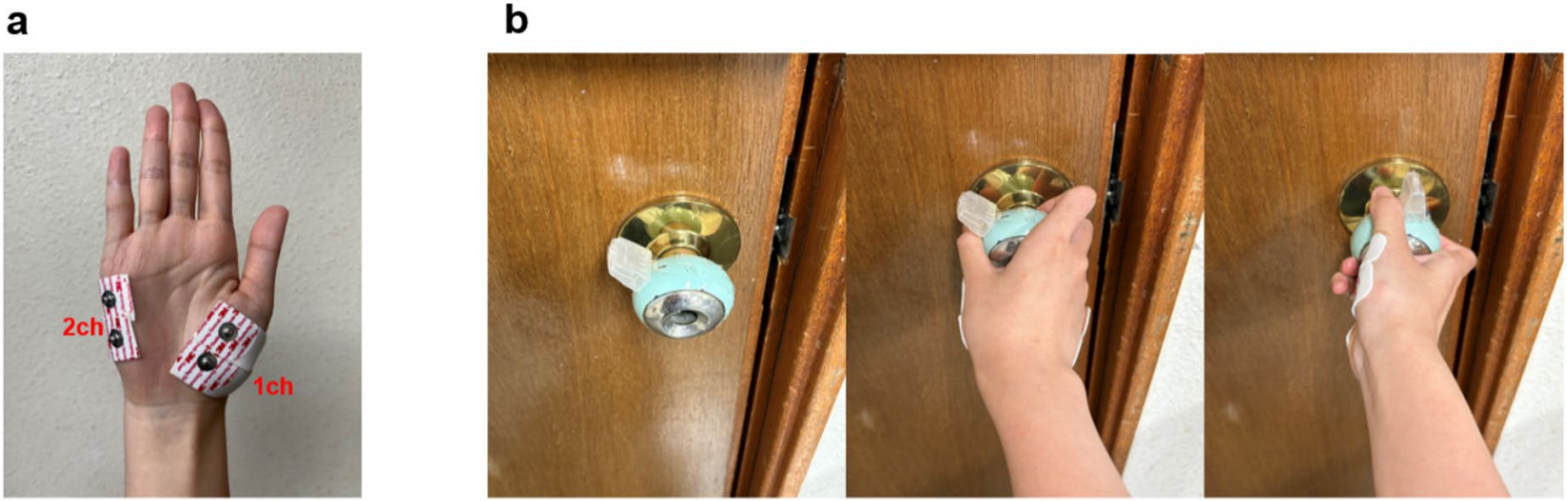
Electrode placement and measurement task, (**a**) sEMG electrode placement location, and (**b**) movements performed for data measurements.

To stably measure electromyographic signals generated during finger flexion and handle manipulation, this study standardized the electrode attachment locations and measurement conditions with reference to the SENIAM and IEC 63203-403-1 ED1 guidelines (Fig. 3). Figure 3(a) shows the sEMG sensor attachment locations. Electrodes were attached to the abductor pollicis brevis (channel 1) and the abductor digiti minimi (channel 2) of the right hand, and the reference electrode was placed on the ulnar styloid process. These muscles exhibit clear activation during finger flexion and handle grasping movements and can effectively reflect user-specific muscle activation patterns according to hand movements.

Figure 3(b) shows the actual handle manipulation task used in the experiment. Each participant repeatedly performed the action of grasping and rotating a round doorknob a total of 50 times, and to minimize variability caused by individual differences in grasping habits, the position of the thumb was pre-specified and fixed. Through this, unnecessary changes in finger position or differences in the axis of rotation that could occur during handle manipulation were suppressed, allowing user-specific electromyographic characteristics to be reflected more consistently.

The sEMG signals were collected at a sampling frequency of 1,000 Hz, and each action consisted of a 1-second grasping phase, a 1-second rotation phase, and a 1-second stationary phase, thereby securing a total of 3 s of effective signal duration. Accordingly, each trial had a length of 3 s × 1,000 Hz along the time axis and was structured as tensor data in the form of 3 × 2 × 1000, including two measurement channels. This data structure was designed to be suitable for subsequent time–frequency transformation and input into the deep learning model.

The entire collected dataset was divided into training and testing data at a ratio of 8:2, and the data were arranged so that the distribution of data for each user was balanced during the split. This experimental design aims to prevent overfitting during model training and to more reliably evaluate user identification performance on data that were not used for training.

The data used in this study were collected after obtaining approval from the Institutional Review Board (IRB) of Kyungpook National University Hospital, in accordance with ethical guidelines (IRB number: KNUH 2025). The research was conducted in accordance with the Declaration of Helsinki and applicable regulations. Written informed consent was obtained from all participants.

## Results

### Comparison between the baseline models and the proposed model

To validate the performance of the proposed DenseNet161 model, comparative experiments were conducted with various baseline classifiers under the same dataset and preprocessing pipeline conditions. The comparison targets included 1D CNN and 2D CNN used in previous sEMG-based user identification studies^17^, and the ResNet18^31^ model, which enables stable training through a residual connection structure, was also evaluated. In addition, to consider temporal dependencies, CNN–BiLSTM^22^ and CNN–LSTM–Attention^23^ architectures were included, and ConvNeXt^32^, which has demonstrated strong performance in the field of computer vision, was added as a baseline model. Furthermore, a broad comparison was conducted with recent CNN-based models having diverse structural characteristics, including Tri-CCNN^25^ with a multi-path convolution structure and E2CNN^24^ utilizing a lightweight feature combination structure. The performance of all models was evaluated based on test accuracy and F1-score (Table 3).

**Table 3:** Classification performances of the proposed model and baseline models.

Model	Test accuracy (%)	F1-score (%)
1D CNN	86.63	86.26
2D CNN	91.58	91.63
ResNet18	88.11	88.04
CNN-LSTM-Attention	88.21	88.24
ConvNeXt	81.16	81.19
Tri-CCNN	81.79	71.78
E2CNN	88.42	88.26
CNN-BiLSTM	85.58	85.50
DenseNet161 (proposed)	94.00	93.99

The 1D CNN showed an accuracy of 86.63% and an F1-score of 86.26%, while the 2D CNN achieved an accuracy of 91.58% and an F1-score of 91.63%, confirming that performance improved when utilizing time–frequency representations. ResNet18 recorded an accuracy of 88.11% and an F1-score of 88.04%, and the CNN–LSTM–Attention and CNN–BiLSTM models exhibited performance levels of approximately 88.2% and 85.6%, respectively. ConvNeXt, E2CNN, and Tri-CCNN achieved accuracies of 81.16%, 88.42%, and 81.79%, respectively.

In contrast, the proposed DenseNet161 model recorded an accuracy of 94.00% and an F1-score of 93.99%, showing the best performance among all baseline models included in the comparison. Compared to 1D CNN, this represents improvements of 7.37% and 7.73% in accuracy and F1-score, respectively; compared to 2D CNN, improvements of 2.42% and 2.36%; and compared to ResNet18, improvements of 5.89% and 5.95%, respectively. Even when compared with CNN–LSTM–Attention and CNN–BiLSTM architectures that explicitly model temporal dependencies, DenseNet161 consistently maintained higher accuracy and F1-scores. Moreover, a clear performance advantage was also confirmed in comparison with the latest CNN architecture, ConvNeXt. These results suggest that the dense connectivity structure of DenseNet effectively reuses features learned from previous layers, which is advantageous for more precisely preserving and extracting subtle user-specific patterns from sEMG signals represented in the time–frequency domain.

**Fig. 4:**
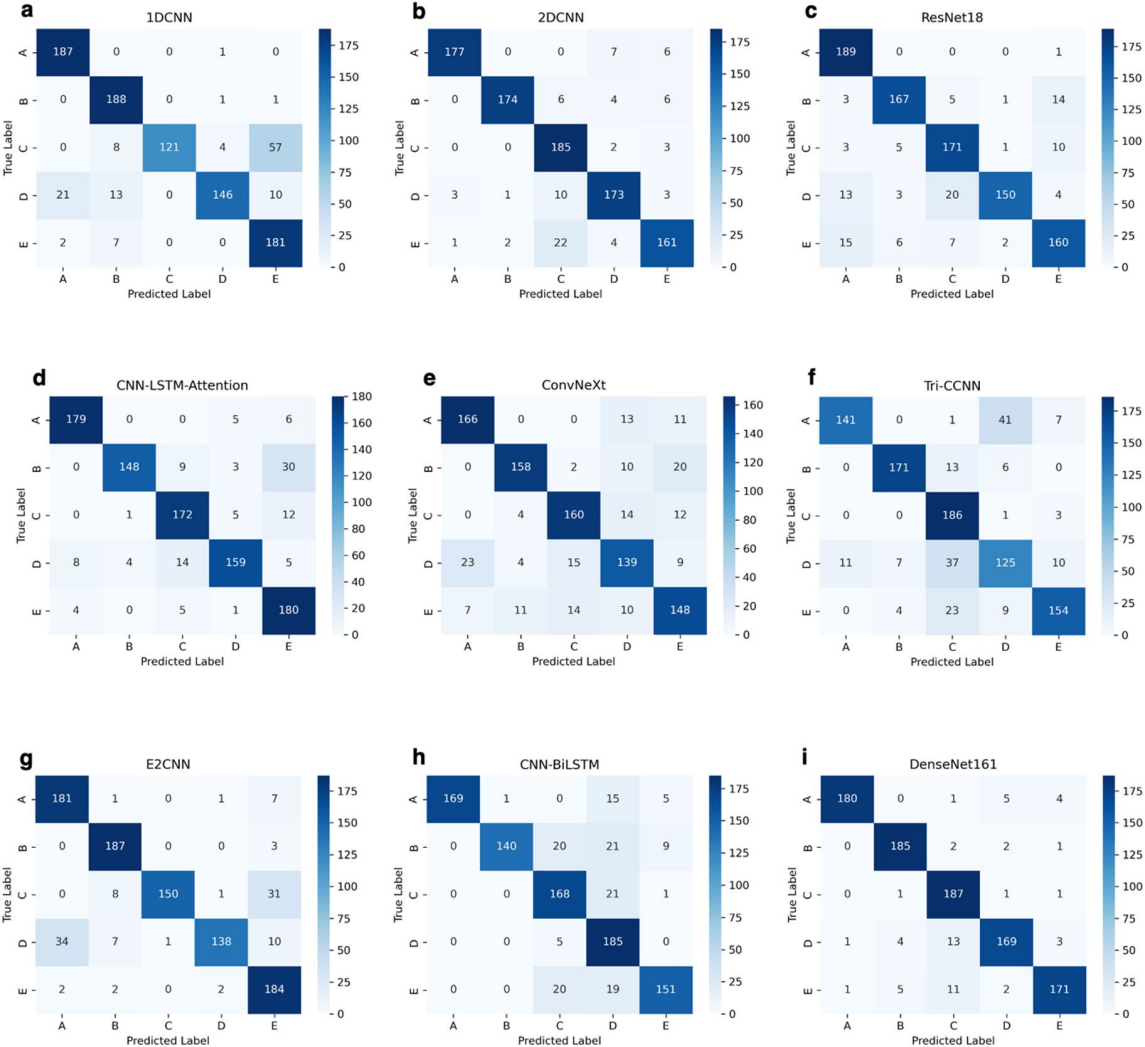
Classification results confusion matrices for (**a**) 1D CNN, (**b**) 2D CNN, (**c**) ResNet18, (**d**) CNN-LSTM-Attention, (**e**) ConvNeXt, (**f**) Tri-CCNN, (**g**) E2CNN, (**h**) CNN-BiLSTM, (**i**) DenseNet161(the proposed model).

The following presents the test results of each comparative model and the proposed DenseNet161 in the form of confusion matrices for a total of five classes (A–E) (Fig. 4). Each class represents one of the five participants. In all confusion matrices, the diagonal components were dominant overall, indicating generally satisfactory classification performance; however, distinct differences in misclassification patterns among classes were observed depending on the model.

In the case of 1D CNN and 2D CNN, relatively high misclassification occurred between classes C and D, and between classes C and E, suggesting that time-domain or simple two-dimensional representations alone are insufficient to fully distinguish subtle user-specific patterns between certain classes. ResNet18, by securing training stability through residual connections, strengthened the diagonal components and reduced inter-class confusion compared to 1D and 2D CNN; however, a certain level of misclassification still remained between classes D and C, and between classes E and C.

Although CNN–LSTM–Attention and CNN–BiLSTM models incorporating recurrent structures reflect temporal dependencies, persistent misclassification occurred in specific class pairs, preventing balanced performance across all classes. Similarly, the latest CNN architecture ConvNeXt showed high classification accuracy in some classes but exhibited relatively larger misclassification due to insufficient capture of distribution imbalance among classes and fine differences in time–frequency patterns.

In contrast, the proposed DenseNet161 recorded the highest diagonal components across all five classes, and notably reduced misclassification between classes C–D and C–E that had been frequently observed in previous models. This is interpreted as a result of the dense connectivity structure of DenseNet effectively reusing features extracted from previous layers, thereby contributing to preserving and enhancing subtle user-specific patterns within sEMG signals transformed into the time–frequency domain without loss. The confusion matrix analysis clearly demonstrates that the proposed model not only improves overall accuracy but also possesses superior performance in terms of inter-class discriminative capability.

### Cross-validation results for the proposed model

**Fig. 5:**
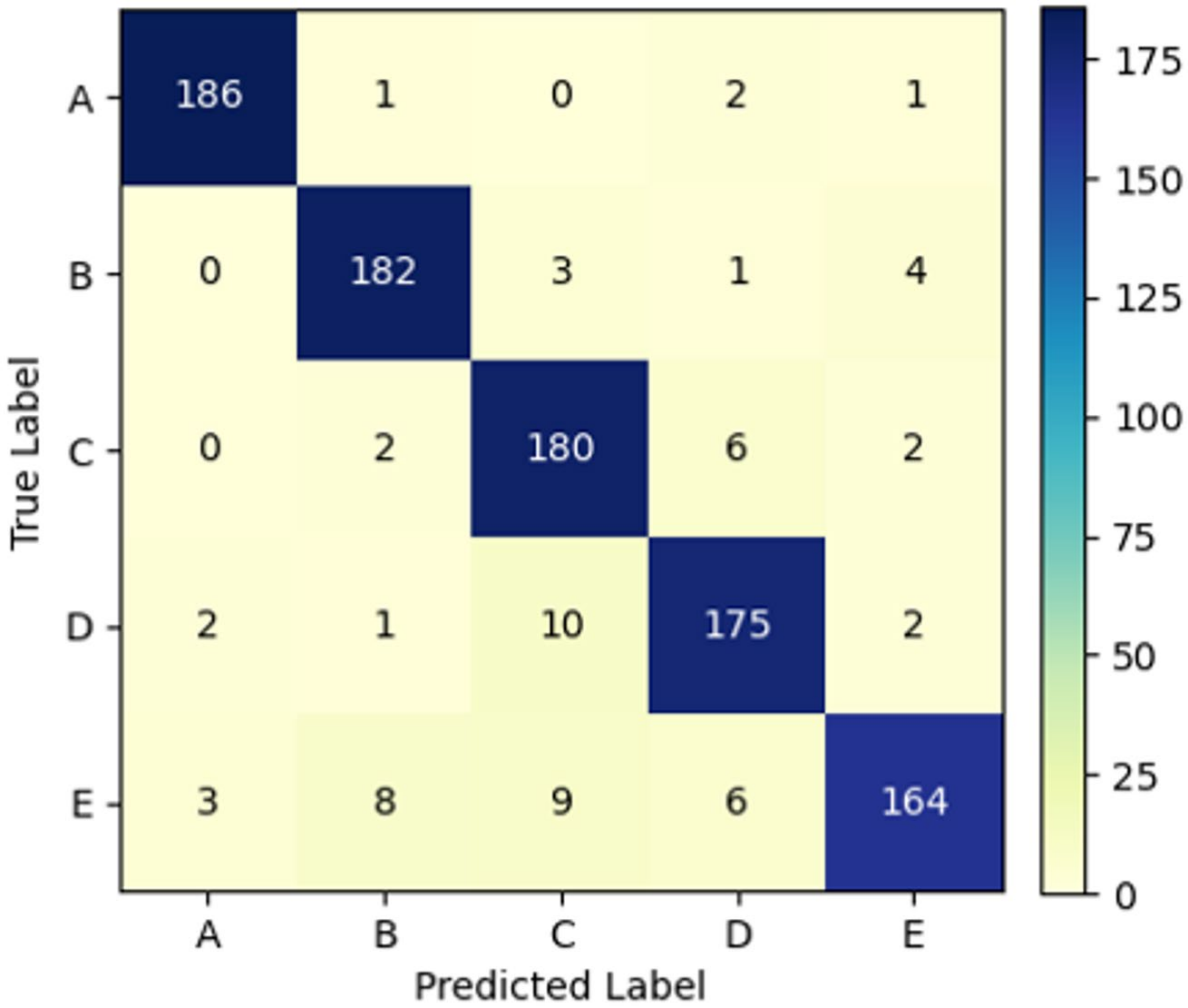
5-Fold best model confusion matrix.

These are the results of the 5-fold cross-validation and the final test performance of the proposed DenseNet161 model (Fig. 5). In the 5-fold cross-validation, the average accuracy was 91.66% with a standard deviation of$\:\pm\:$2.78%, and the average F1-score was 91.64%$\:\pm\:$2.80%. The relatively small standard deviation indicates that performance variation due to data splitting was not large, suggesting that the proposed model maintains stable classification performance under different training–validation split conditions. In particular, the fact that the average values of accuracy and F1-score were at nearly the same level indicates that balanced predictions between precision and recall were achieved without bias toward a specific class, even under class-imbalanced conditions.

In addition, when the model selected based on the best-performing fold during the cross-validation process was applied to an independent test set, it achieved an accuracy of 93.37% and an F1-score of 93.35%. This represents an improvement of approximately 1.7% compared to the average cross-validation performance, demonstrating that the proposed model is not specialized only to the training data and maintains stable predictive performance even on independent data.

### Comparison of computational complexity between baseline models and the proposed model

This section presents a comparison of the training time, inference time, and number of trainable parameters of the models evaluated in this study^33^ (Table 4). Lightweight models such as 1D CNN and Tri-CCNN required approximately 144 s and 245 s of training time, respectively, and had very small numbers of parameters. Although they were superior in terms of computational efficiency, they exhibited relatively limited classification performance. E2CNN also maintained a relatively small number of parameters and short inference time while providing a certain level of performance; however, it has limitations in sufficiently modeling complex user-specific patterns.

Although CNN–LSTM–Attention and CNN–BiLSTM models incorporating recurrent structures can reflect temporal dependencies, the performance improvement relative to the increase in training time and number of parameters was limited. This suggests that recurrent structures are not necessarily essential for sEMG-based user identification problems. ResNet18 and ConvNeXt enhanced representational power through deep CNN architectures; however, as the number of parameters increased significantly to approximately 11 million and 27.8 million, respectively, the training and inference costs also became relatively higher.

The proposed DenseNet161 belongs to the models with the largest training time and number of parameters among the compared models, and accordingly showed relatively increased inference time as well. However, this increase in computational cost led to a clear advantage in user identification performance, consistently recording the highest accuracy and F1-score in this study. This is because the dense connectivity structure of DenseNet effectively preserves and reuses subtle user-specific patterns in time–frequency-based sEMG representations, thereby enabling more precise feature learning. Consequently, the proposed model has sufficient practical value in user identification scenarios where identification accuracy and stability are more important than computational efficiency, and suggests that it can be a meaningful choice, particularly in environments requiring high reliability and security.

**Table 4:** Computational complexity of baseline models and the proposed model.

Model	Training time (seconds)	Inference time (seconds)	Number of trainable parameters
1D-CNN	144	2.94	9,589
2D-CNN	601	1.35	4,944,901
Tri-CCNN	245	0.88	1,357
E2CNN	389	1.04	85,369
CNN–LSTM–Attention	549	2.14	372,870
CNN–BiLSTM	549	2.14	1,798,693
ResNet18	1,191	2.76	11,179,077
ConvNeXt	1,423	4.31	27,823,877
DenseNet161(proposed)	6,441	10.49	26,483,045

### Statistical significance test of the proposed model

To verify whether the performance differences between the proposed DenseNet161 model and the baseline models were the result of chance, the Wilcoxon signed-rank test was performed based on the 10-fold cross-validation results of each model^34^. This test is a non-parametric statistical method, which has the advantage of evaluating the statistical significance of differences between paired samples without assuming normality of the performance distribution, and is therefore suitable for cross-validation-based performance comparison. Accuracy and weighted F1-score were used as comparison metrics, and the significance level was set to 0.05 (Table 5).

The proposed DenseNet161 model showed statistically significant performance differences (*p* < 0.05) in comparisons with all baseline models. This indicates that the performance improvement of the proposed model was not the result of a specific data split or chance, but rather was consistently observed across the overall 10-fold cross-validation. Therefore, it was confirmed that the DenseNet161 model provides superior performance compared to existing models in sEMG-based user identification problems at a statistically reliable level.

**Table 5:** Statistical significance test results using Wilcoxon signed-rank test.

Comparison	*p*-value	Significance
Proposed DenseNet161 vs. 2D CNN	*p* < 0.05	Yes
Proposed DenseNet161 vs. ResNet18	*p* < 0.05	Yes
Proposed DenseNet161 vs. CNN–LSTM–Attention	*p* < 0.05	Yes
Proposed DenseNet161 vs. ConvNeXt	*p* < 0.05	Yes
Proposed DenseNet161 vs. Tri-CCNN	*p* < 0.05	Yes
Proposed DenseNet161 vs. E2CNN	*p* < 0.05	Yes
Proposed DenseNet161 vs. CNN–BiLSTM	*p* < 0.05	Yes

## Discussion

Research on user identification using EMG signals has primarily relied on approaches that analyze temporal or spectral features based on signals collected from the forearm. Because sEMG signals exhibit both temporal characteristics and nonlinear variations depending on muscle activation states, one-dimensional signal-based approaches may have limitations in stably distinguishing subtle differences among users. Accordingly, previous studies have attempted to improve identification performance by combining multi-channel sEMG signals collected from the forearm or upper arm with time–frequency feature extraction techniques. Fan et al.^15^ utilized forearm EMG and a Siamese CNN, Gursoy^16^ presented a model combining DWT, EWT, and EMD with CNN, and Kim et al.^17^ applied CQT-based two-dimensional spectrograms to CNN. In addition, Lu et al.^18^ reported user identification performance through a CNN architecture combining CWT and transfer learning. Although these prior studies were effective in feature extraction in the frequency domain, they simultaneously entail limitations such as dependence on multi-channel signals, redundant information among channels, and system complexity when applied in real environments.

In this study, to address these limitations, we experimentally verified whether user identification is possible using only two-channel sEMG signals acquired from the palm rather than the forearm or wrist. In particular, this study extended the user identification problem to a more realistic usage scenario by setting doorknob rotation, a naturally repeated action in real environments rather than an intentionally performed gesture, as the identification input. In this context, the system targets a closed-set user identification problem that determines the performer within a registered user set, and is clearly distinguished from existing authentication-focused studies in that it performs multi-class identification rather than binary classification-based authentication decisions.

The collected palm sEMG signals were preprocessed to remove noise and then transformed into two-dimensional spectrograms in the time–frequency domain by applying CWT. CWT enhances frequency resolution in the low-frequency region and time resolution in the high-frequency region, thereby effectively reflecting the non-stationary characteristics of sEMG signals. Through this, it was possible to simultaneously capture temporal changes in muscle activation and frequency distributions occurring during doorknob rotation, contributing to distinguishing subtle differences in electromyographic signals among users.

From the perspective of model architecture, this study compared various CNN-based models under the same input conditions, and among them DenseNet161 showed the most stable identification performance. While conventional 1D or 2D CNN architectures have the structural characteristic that each convolutional layer utilizes only the output of the previous layer, DenseNet has a dense connectivity structure in which all previous layer features are directly utilized by subsequent layers. This feature reuse mechanism acted advantageously in minimizing the loss of user-specific patterns even with a limited number of channels and relatively short signal segments. As a result, the combination of the proposed CWT-based two-dimensional spectrogram and the DenseNet architecture improved user identification performance by efficiently utilizing multi-dimensional time–frequency features.

In the experimental results, the DenseNet161 model showed superior performance compared to eight baseline models, recording an accuracy of 91.66$\:\pm\:$2.78% and an F1-score of 91.64$\:\pm\:$2.80% based on five-fold cross-validation. In addition, when evaluated based on the fold showing the best performance, it achieved an accuracy of 93.37% and an F1-score of 93.35%. This indicates not only an improvement in average performance but also that inter-user identification stability was generally secured. These results experimentally demonstrate that user identification using only palm sEMG can have sufficient discriminative power.

The approach of this study also has significance from a practical deployment perspective. Palm sEMG-based user identification does not presuppose wearable devices or wireless connections and can be implemented in the form of integrating sensors into physical interfaces. For example, in environments where users directly touch objects with their hands, such as doorknobs, drawer handles, grips of shared equipment, or control parts of specific tools, user identification is possible without a separate recognition procedure. This provides a structure suitable for closed-set identification scenarios targeting a limited user group, such as access control or equipment usage history management.

Meanwhile, this study has several limitations. First, the number of participants was limited, and since data for the same user were collected primarily within a single measurement session, long-term variability due to date changes, muscle fatigue, or changes in skin condition was not sufficiently reflected. Unlike some prior studies that utilized large-scale public datasets such as NinaPro, this study was conducted on a relatively small, self-collected dataset, which may limit the generalizability of the proposed model. Because the study focused on the single task of doorknob rotation, additional validation is required regarding performance stability when extended to environments with different handle types, materials, or manipulation resistance. Furthermore, since DenseNet161 has relatively high computational complexity, consideration of model lightweighting is required for application in real embedded environments or low-power systems.

Overall, the present study contributes to the literature by showing that palm sEMG generated during doorknob rotation can support closed-set user identification in a practical everyday setting. Unlike previous wearable- or gesture-centered approaches, the proposed method suggests a contact-based, interface-integrated identification framework that operates without wrist/forearm sensors or wireless pairing. This distinction highlights the novelty and practical relevance of the proposed approach.

Future work will verify generalization performance through experiments including a larger number of users and multi-session data, and expand experiments to various object manipulation tasks to evaluate robustness against environmental changes. In addition, lightweight CNN architectures or model compression techniques will be applied to develop a user identification system suitable for real-time deployment.

## Data Availability

The datasets generated during and/or analysed during the current study are available in the sea3551/palm-sEMG-doorknob-filtered repository, https://github.com/sea3551/palm-sEMG-doorknob-filtered.git.
